# Ki-67 Expression in Hydatidiform Moles and Hydropic Abortions

**DOI:** 10.5812/ircmj.5348

**Published:** 2013-07-05

**Authors:** Alireza Khooei, Fatemeh Atabaki Pasdar, Alireza Fazel, Mahmoud Mahmoudi, Mohammad Reza Nikravesh, Mohammad Khaje Delui, Bagher Pourheydar

**Affiliations:** 1 Department of Pathology, Imam Reza Hospital, Mashhad University of Medical Sciences, Mashhad, IR Iran; 2 Department of Anatomical Sciences, Urmia University of Medical Sciences, Urmia, IR Iran; 3 Department of Anatomy and Cell Biology, Mashhad University of Medical Sciences, Mashhad, IR Iran; 4 Immunology Research Center, Bu Ali Research Institute, Mashhad University of Medical Sciences, Mashhad, IR Iran; 5 Department of Medical Ethics, Mashhad University of Medical Sciences, Mashhad, IR Iran

**Keywords:** Hydatidiform Mole, Abortion, Immunohistochemistry, Ki-67 Antigen

## Abstract

**Background:**

Differential diagnosis of hydatidiform moles from non-molar specimens as well as their sub-classification such as complete and partial hydatidiform moles are important for clinical management and accurate risk assessment for persistent gestational trophoblastic disease, but diagnosis based solely on histomorphology suffers from poor interobserver and intraobserver reproducibility.

**Objectives:**

This study was undertaken to determine whether the expression of Ki-67 protein could differentiate these entities.

**Materials and Methods:**

We performed Ki-67 immunohistochemical staining in 19 molar (8 partial and 11 complete moles) and 10 non-molar (hydropic abortions) formalin-fixed, paraffin-embedded tissue samples. Ploidy analysis using flow cytometry had confirmed diploidy in hydropic abortions and complete moles and triploidy in partial moles.

**Results:**

Ki-67 immunoreactivity was assessed in villous cytotrophoblasts, syncytiotrophoblasts and stromal cells. Positive cells were found to be restricted mostly to the villous cytotrophoblasts, while syncytiotrophoblasts showed an absence of immunostaining for Ki-67, and occasional weak nuclear staining was seen in the stromal cells. There was a significant difference in Ki-67 immunoreactivity of cytotrophoblastic cells between hydropic abortions and complete moles (P < 0.001), hydropic abortions and partial moles (P = 0.002) and also between complete and partial moles (P < 0.001). On the other hand, there is significant overlap in the Ki-67 immunoreactivity between complete and partial moles (++ staining category) and between partial moles and hydropic abortions (+ staining category).

**Conclusions:**

Despite the significant differences , Ki-67 immunostaining could not be helpful in distinguishing molar placentas from hydropic abortions as well as partial from complete hydatidiform moles, because there are considerable overlaps between results in different categories.

## 1. Background

Hydatidiform moles are most common form of gestational trophoblastic disease that result from abnormal fertilization and characterized by hydropic swelling of placental villi and trophoblastic hyperplasia (1). They are categorized into partial and complete forms based on morphologic, genetic and clinical features. The incidence of molar gestation varies geographically, being highest in Asian countries (2). Hydatidiform moles have attracted much attention, because approximately 10-30% of complete moles and 0.5-5% of partial moles progress to persistent trophoblastic diseases (3). Despite well-described histopathologic criteria, the distinction of hydropic abortion from hydatidiform mole, and complete hydatidiform mole from partial hydatidiform mole remain a problem because of interobserver and intraobserver variability (4, 5); Especially that during early pregnancy the diagnostic criteria are subtly different from the classical pathological features (6). These errors can be significantly reduced by ploidy analysis. Ploidy evaluation by flow cytometry has been successfully used for fresh and fixed tissues, and has become widely accepted as a reliable test for ploidy (7). It can distinguish diploid complete hydatidiform moles (androgenetic diploidy) or hydropic abortions (biparental diploidy) from triploid partial hydatidiform moles (diandric monogynic triploidy), however this method cannot distinguish diploid complete moles from diploid non-molar products of conceptions. On the other hand, some non-molar specimens can have digynic triploidy (2 maternal and 1 paternal chromosome complements) (8). Immunohistochemical methods are relatively simple alternative to the more complex techniques. One of the advantages of these methods is the ability to apply them retrospectively to sections of routinely formalin-fixed, paraffin-embedded tissuues. Another advantage is that there is no need for expensive or sophisticated equipments. The fact that the Ki-67 protein is present during all active phases of the cell cycle (G1, S, G2 and mitosis), but is absent from quiscent or resting cells (G0), makes it an excellent marker for reflection of the tissue proliferation compartment (9) and thus could be of value in studying the biologic behavior of gestational trophoblastic diseases.

## 2. Objectives

The aim of this study is to evaluate the expression of Ki-67 in molar pregnancies (complete and partial hydatidiform moles) and non-molar (hydropic spontaneous abortions), also to assess the values of this marker in differential diagnosis of these entities.

## 3. Materials and Methods

### 3.1. Case Selection

Formalin-fixed, paraffin-embedded gestational products from 29 patients, including 11 complete hydatidiform moles, 8 partial hydatidiform moles and 10 hydropic spontaneous abortions diagnosed in the Imam Reza and Qhaem Departments of pathology, Mashhad University of Medical Sciences were gathered. Gestational age ranged from 8 to 16 weeks (mean, 11.6 weeks). Tissue sections of the specimens were stained with routine hematoxylin-eosin and histopathologically reviewed by the pathologist, using published criteria (10) for the confirmation of diagnosis. Ploidy analysis using flow cytometry was performed and confirmed diploidy in spontaneous abortions and complete moles, and triploidy in partial moles.

### 3.2. Flow Cytometry

Flow cytometric DNA analysis was performed on formalin-fixed, paraffin-embedded tissue blocks. The selection criterion for the blocks was the presence of both placental and maternal (decidual) tissue in approximately such amounts that representative DNA histograms could be anticipated. Maternal tissue had to be present as the internal diploid control. One 50 μm section of each block was placed in 10 ml glass centrifuge tubes and dewaxed using two changes of xylene, 3 ml for 10 min at room temperature, and then rehydrated in a sequence of 3 ml of 100%, 95%, 75%, and 50% ethanol for 10 min each at room temperature with centrifugation and decantation of the supernatant after each step. The tissues were then washed twice in distilled water and re-suspended in pepsin solution (1 mL of 0.05% pepsin in 0.9% NaCl, pH 1.5) at 37°C for 45-60 minutes with intermittent mixing using a vortex. The reaction was stopped with cold PBS and the samples were washed twice with phosphate buffered saline (PBS).The resulting cell suspension was washed twice with PBS. After addition of RNase to remove any nuclear or residual cytoplasmic RNA, and propidium iodide, ploidy was determined by flow cytometry using facscalibur flow cytometer (Becton-Dickinson). Histograms were generated from analysis of 10000 nuclei and displayed as linear fluorescence.(6, 7).

### 3.3. Immunohistochemistry

5μm thick sections were cut and incubated for 60 min at 60ºC, then the sections deparaffinized in xylene and rehydrated in a descending ethanol series. Endogenous peroxidase activity was blocked by a 20 minute treatment with three percent hydrogen peroxidase in phosphate-buffered saline (PBS). The slides were then washed twice in PBS, pH 7.4 and subsequently transferred to retrieval buffer (10-Mm sodium citrate buffer, pH 6.0) and heated in a microwave oven (at a power of 700 W). The slides were left to cool at room temperature, then were incubated with mouse monoclonal antibody for 30 min at room temperature (Ki-67: prediluted (ready to use), Clone MIB1, N1633, Dakocytomation, Glostrup, Denmark). Later the sections were rinsed in PBS and incubated with polymer-based Envision (Dako Cytomation, Glostrup, Denmark). The chromogenic reaction was performed by 3, 3-diaminobenzidine (DAB), (Dako Cytomation, Glostrup, Denmark). The sections were then counterstained with Mayer̛s hematoxylin. The sections of a reactive lymph node were used as a positive control for KI-67, negative controls were stained by skipping primary antibody incubation. Evaluation of protein expression was carried out. All immunostained sections were independently examined by the same two observers with a ×400 objective under the light microscope (Olympus BX-51, Olympus, Tokyo, Japan), while they did not know about the slide diagnosis, therefore the analysis was double-blind. Immunoexpression analyses for villous cytotrophoblasts, syncytiotrophoblasts and stromal cells, commenced from the field with most staining, separately by counting 100 cells of each population per slide(11-14). The immunoreactivity was assessed as: 0 (no stained cells), + (≤ 25 % positive cells), ++ (26-50 % positive cells) and +++ (more than 50 % positive cells).

### 3.4. Statistical Analysis

Statistical analyses were conducted using Kolmogorov-Smirnov, ANOVA and Tukey’s HSD tests. The results were expressed as mean ± SD. The differences were considered statistically significant at a P value less than 0.05. All statistical tests were performed using SPSS 11.5 (SPSS Inc., Chicago, IL, USA) software.

## 4. Results

**Figure 1.**
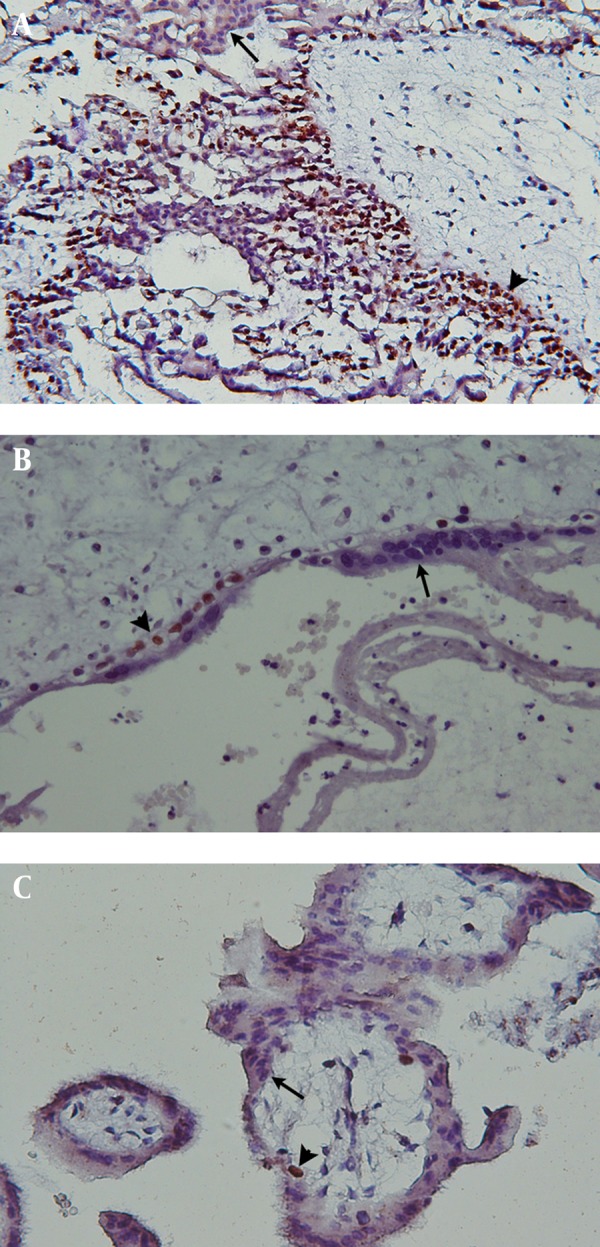
Immunoreactivity With Ki67 in Complete Hydatidiform Mole, A) Partial Hydatidiform Mole, B) Hydropic Spontaneous Abortion, C) Tissues were counterstained with Mayer’s hematoxylin, the syncytiotrophoblasts and cytotrophoblasts are indicated by arrows and arrowheads, respectively Immunoreactivity is confined to the nuclei of the cytotrophoblasts and the syncytiotrophoblasts are negative in all cases. Magnification was ×200 in (a) and × 400 in (b, c).

Ki-67 immunoreactivity was assessed in villous cytotrophoblasts, syncytiotrophoblasts and stromal cells. Positive cells were found to be restricted mostly to the villous cytotrophoblasts, while syncytiotrophoblasts showed an absence of immunostaining for Ki-67, and occasional weak nuclear staining was seen in the stromal cells. There was a significant difference in Ki-67 immunoreactivity with cytotrophoblastic cells between complete hydatidiform moles (Figure 1 a) and partial hydatidiform moles (Figure 1 b) (P < 0.001), complete hydatidiform moles and hydropic abortions (Figure 1 c) (P < 0.001) and also between hydropic abortions and partial hydatidiform moles (P = 0.002). 

On the other hand, there is significant overlap in the Ki-67 immunoreactivity between complete and partial moles (++ staining category) and between partial moles and hydropic abortions (+ staining category). The results of statistical analyses are summarized in (Tables 1, 2, 3).

**Table 1.** Ki-67 Labeling Index (% of Positively Stained Nuclei/Total Number of Nuclei Counted)

TYPE of Lesion	Cytotrophoblasts	Syncytiotrophoblasts	Stromal Cells
**CHM ^a^, n = 11**	55.73 ± 16.90	0	3.45 ± 1.03
**PHM , n = 8** ^**a**^ **, n = 8**	26.25 ± 9.92	0	3.37 ± 1.41
**HA , n = 10** ^**a**^ **, n = 10**	6.80 ± 2.10	0	2.40 ± 1.50

^a^ Abbreviations: CHM, complete hydatidiform mole; PHM, partial Hhydatidiform mole; HA, hydropic abortion

**Table 2.** Distribution of Ki-67 Immunoreactivity, (%)

Types of lesion	Cytotrophoblasts				Syncytotrophoblasts				Stromal Cells			
	0 ^a^	+ ^a^	++ ^a^	+++ ^a^	0	+	++	+++	0	+	++	+++
**CHM ^b^**	0	0	5	6	11	0	0	0	0	11	0	0
**PHM ** ^**b**^	0	3	5	0	8	0	0	0	0	8	0	0
**HA ** ^**b**^	0	10	0	0	10	0	0	0	1	9	0	0

^a^ 0 (no stained cells), + (≤ 25 % positive cells), ++ (26-50 % positive cells), +++ (> 50 % positive cells)

^b^Abbreviations: CHM, complete hydatidiform mole; PHM, partial hydatidiform mole; HA, hydropic abortion

**Table 3.** Results of Statistical Analysis to Compare Ki-67 Expression between Groups

Types of lesions to be separated	Ki-67 Labelling Index		
	Cytotrophoblasts	Syncytiotrophoblasts	Stromal Cells
**CHM ^a^, PHM^a^**	P < 0.001	n ^b^	P = 0.99
**CHM, HA ** ^a^	P < 0.001	n ^b^	P = 0.307
**PHM, HA**	P = 0.002	n ^b^	P = 0.447

^a^ Abbreviations: CHM, complete hydatidiform mole; PHM, partial hydatidiform mole; HA, hydropic abortion

^b^ n: no statistics are computed

## 5. Discussion

The histologic separation of spontaneous abortions especially those with hydropic changes from partial moles and of partial from complete moles may be difficult. Although diagnostic criteria are established, there is considerable intra and interobserver variability when using gross and microscopic findings alone. All authors agree on the risk of molar disease to developing persistent gestational trophoblastic tumors and most of them have emphasized the importance of some ancillary techniques as cytometry, molecular genotyping, histochemistry and immunohistochemistry to improve diagnosis (7, 15, 16). The value of immunohistochemical analysis of paternally imprinted, maternally expressed p57 gene for improving the diagnosis of hydatidiform moles has been demonstrated in a number of recent studies, (16, 17). However, p57 immunohistochemistry can identify complete hydatidiform moles (androgenetic diploidy) by the lack of p57 expression but cannot distinguish partial hydatidiform moles (diandric monogynic triploidy) from non-molar (biparental diploidy) specimens. Immunohistochemical determination of cell proliferation associated antigens has provoked the interest of histopathologists in recent years. One of the most widely used reagents in this field is the antibody Ki-67. This reacts with a nuclear non-histone protein of 395 and 345 kilodaltons present in all active parts of the cell cycle (G1,S,G2 and M), but is absent in G_0_ (9). In this study, immunoreactivity for Ki-67 in hydropic abortions and hydatidiform moles were largely confined to cytotrophoblasts. This pattern is in accordance with previous studies which have identified the cytotrophoblast as the active germinative zone, based on results obtained by autoradiography, total organ DNA analysis, flow cytometry, morphometric analysis and studies on proliferating cell nuclear antigen.(18, 19). On the other hand, in this study, the Ki-67 labeling index in cytotrophoblastic cells significantly differed between the molar and non-molar specimens, as well as between complete and partial moles. This is consistent with previous studies done by Kale et al, 20 and Erfanian et al (12).

In addition, Ostrzega et al ± 10.0 % in hydropic abortions. Schammel and Bocklage reported that Ki-67 immunostaining differed significantly between the molar and non-molar placentas, but did not allow distinction of partial from complete hydatidiform moles (14). Conversely, Cheville et al reported that Ki-67 may be useful in separating complete moles from partial moles but not partial moles from hydropic abortions (20). Such variations may be related to technical factors such as the time of fixation, because the tissues used in these studies were archival material with no standardised fixation time, or different detection methods may affect the results of immunohistochemistry; furthermore it may be due to the difference in gestational age of samples. As described in most of previous studies (14, 20-23), there was no immunostaining for Ki-67 in the nuclei of syncytiotrophoblasts, however Erfanian et al reported that Ki-67 expression was observed in syncytiotrophoblastic cells of abortion, partial hydatidiform mole, complete hydatidiform mole and choriocarcinoma (12). Contrary to cytotrophoblast which is the trophoblastic stem cell, syncytiotrophoblast is the terminally differentiated cell that produces most of the placental hormones and regulates the diffusion of oxygen, co_2_ and other nutrients between the mother and fetus (24). Despite the significance of differences, Ki-67 immunostaining could not be helpful in distinguishing molar placentas from hydropic abortions as well as partial from complete hydatidiform moles, because there are considerable overlaps between results in different categories.
